# A Personalized Anisotropic Margin for Cervical Cancer Radiation Therapy Under the Guidance of Daily Iterative Cone-Beam Computed Tomography (iCBCT)

**DOI:** 10.7759/cureus.69029

**Published:** 2024-09-09

**Authors:** Haonan Xiao, Qiufen Guo, Junjie Ma, Jian Chen, Peng Xie, Yong Yin

**Affiliations:** 1 Department of Radiation Oncology and Physics, Shandong Cancer Hospital and Institute, Shandong First Medical University and Shandong Academy of Medical Sciences, Jinan, CHN; 2 Department of Radiation Oncology and Physics, Shandong Provincial Key Medical and Health Laboratory of Pediatric Cancer Precision Radiotherapy, Shandong Cancer Hospital, Jinan, CHN; 3 Department of Radiation Oncology, Shandong Cancer Hospital and Institute, Shandong First Medical University and Shandong Academy of Medical Sciences, Jinan, CHN; 4 Department of Nuclear Science and Technology, School of Nuclear Science and Technology, University of South China, Hengyang, CHN

**Keywords:** cervical cancer, intrafraction motion, motion management, online art, varian ethos

## Abstract

Online adaptive radiation therapy (ART) eliminates interfraction uncertainties by adaption before each treatment session. However, intrafraction motions still exist and could become more severe due to long treatment time. Large isotropic margins can ensure clinical target volume (CTV) coverage but at the cost of more organs at risk damage. In this study, we proposed a novel personalized anisotropic margin search algorithm for cervical cancer radiation therapy under the guidance of daily iterative cone-beam computed tomography (iCBCT) to find the optimal margin values for each patient, which achieves the smallest possible planning target volume (PTV) and maintains CTV coverage. Twenty-two online Ethos ART treatment sessions were included for analysis. Two iCBCT scans were taken in each session. The first one (iCBCT_1_) was taken after positioning, and the second one (iCBCT_2_) was taken before beam delivery. Corresponding CTV_1_ and CTV_2 _were contoured in the two scans. In each session, minimal isotropic margins were first searched by iteratively increasing the magnitude until the resulting PTV_iso_ covers 99% of CTV_2_. Afterward, the margin values in all six directions were decreased iteratively until CTV_2_ coverage was smaller than 99% to get the personalized margin and target volume PTV_int_. In addition, the uterus was considered separately, and different margins were found for it and the remaining CTV, respectively, to reduce the target volume of PTV_sep _further. PTV_iso_, PTV_int_, and PTV_sep_ were compared in terms of CTV_2_ coverage and absolute volume. The algorithm successfully generated PTV_iso_, PTV_int_, and PTV_sep_ for all online ART treatment sessions. The mean ± SD values for PTV_iso_^5mm^, PTV_iso_^10mm^, PTV_iso_^15mm^, PTV_int_, and PTV_sep_ were 1,074.0 ± 78.1, 1,519.5 ± 100.4, 2,006.4 ± 122.5, 929.3 ± 73.4, and 845.1 ± 72.5 mL, respectively. The volume difference between PTV_int_ and PTV_sep_ was significant (p < 0.001). All the PTVs ensured an average coverage larger than 99%, and the differences between any two PTVs were insignificant. This study proposed a novel personalized anisotropic margin search algorithm for cervical cancer online ART. Compared to the conventional 5 or 10 mm isotropic margins, the personalized anisotropic margin reduced PTV volume by 13.5% and 38.8%, respectively; if the uterus was considered separately, the volume can be further reduced by 21.3% and 44.3%, respectively, while CTV coverage was still maintained. This algorithm could reduce target volume and potentially spare normal tissue better than isotropic margin expansion.

## Introduction

Radiation therapy (RT) has been a major treatment for cancers, which provides good local rates and comparable prognosis with surgery in many cancer sites [1]. In a typical RT workflow, the patient's anatomy is represented by computed tomography (CT) images and magnetic resonance imaging (MRI) images, if available, based on which an RT plan is made to focus radiation beams on the target volume and spare normal healthy tissues as much as possible [2]. The RT plan is designed once and is usually utilized for 30-40 daily treatment fractions. However, the anatomy does not keep static during the treatment course, and changes can happen in the shape, size, and position of the target and organs at risk (OARs) [3]. The changes can be in the order of centimeters [4], which may lead to compromised tumor control and/or increased normal tissue complication.

To account for the daily anatomical changes, margins are usually applied to clinical target volumes (CTVs) and expanded to planning target volumes (PTVs) for treatment planning [5]. PTVs tolerate anatomical variations during the treatment course and reduce the probability of the target missing but at the cost of increasing irradiated volume. In addition, the CTV-to-PTV margins are population-based and do not account for patient-specific variations, which may be insufficient for some patients and excess for others.

Adaptive radiation therapy (ART) was introduced to address personalized variations in the late 1990s, characterized as “a closed-loop radiation treatment process where the treatment plan can be modified using a systematic feedback of measurements with the intention to improve radiation treatment by systematically monitoring treatment variations and incorporating them to reoptimize the treatment plan early on during the course of treatment” [6]. Although ART holds excellent potential for anatomical variation addressing, ART techniques were mostly offline at the beginning, which requires repetitive RT processes, including resimulation, recontouring, and replanning between treatment fractions [7,8]. Online ART, which needs those adjustments right before beam delivery, is even more challenging for efficiency and has long been considered difficult to implement. With recent scientific and technical innovations, the Varian Ethos system (Varian Medical Systems, Palo Alto, CA) can integrate artificial intelligence-powered autosegmentation and autoplanning in the online ART process. This allows for delivering a reoptimized daily plan based on the anatomy reflected in the high-quality iterative cone-beam computed tomography (iCBCT) images.

As online ART has addressed interfraction uncertainties by adaption before each fraction, including positioning error and patient body weight loss, CTV-to-PTV margins can focus only on intrafraction motions and, therefore, be reduced [9,10]. Some studies have been conducted on the margin shrinkage of Ethos-based online ART treatment, and their results show that online ART can maintain target coverage with significantly decreased irradiated volume and related OAR doses, holding great potential for reducing normal tissue complications [10-12]. For example, Dohopolski et al. compared conventional image-guided radiation therapy (IGRT) plans and online ART plans in head and neck cancer treatment, and their findings suggest that ART plans of 1 mm CTV-to-PTV margins achieved comparable target coverage with IGRT plans of 5 mm margins; in addition, the doses to all OARs were significantly reduced in ART plans except for larynx and post-arytenoid and cricoid space [13]. The intrafraction motion in the pelvic region is larger than the head and neck region, and Yen et al. found that a uniform 5 mm CTV-to-PTV margin was adequate to cover the intrafraction motion. They ensured a CTV coverage of over 98% [14].

Although many studies have been conducted on online ART margin shrinkage, they mostly investigated uniform margins in all six directions, namely superior, inferior, left, right, anterior, and posterior. The motion of the targets, however, is not always isotropic, and a uniform margin may include excess healthy tissues to cover motions in only a few directions [15,16]. Mahantshetty et al. found that for the uterine fundus, the motion amplitude could reach 12 mm in the superior direction but halved in others [17]. Kishigami et al. also demonstrated the effect of anisotropic margins and identified the major motions that happened in the uterus region [18]. Therefore, anisotropic margins that account for the motion differences of each subvolume of the target are recommended [19,20].

In this study, we developed an algorithm to search personalized anisotropic margins for cervical cancer radiation treatment. We monitored the first several treatment fractions of the patient and contoured the regular CTV and uterus (CTVu) separately on the session iCBCT images. The margin was determined for the following fractions afterward. Retrospective analysis was performed to compare the anisotropic margins to regular margins in terms of CTV coverage and PTV volume.

## Case presentation

Patient information

One cervical cancer patient from Shandong Cancer Hospital was included in this study. The patient, a 74-year-old woman, has been diagnosed with cervical squamous cell carcinoma as confirmed by a cervical biopsy. Combined with the pretreatment pelvic MRI and gynecological oncologist consultation, the International Federation of Gynecology and Obstetrics stage was determined to be IIB. The patient had several medical comorbidities, including grade 3 extremely high-risk hypertension, type 2 diabetes, and post-traumatic knee disorder. Due to the patient's poor physical condition and inability to tolerate synchronous chemoradiotherapy, radiotherapy alone was selected as the primary treatment option. The prescription dose for external beam radiotherapy was 50.4 Gy/28 Fx, followed by CT-guided 3D brachytherapy of 30 Gy/6 Fx. The external beam radiotherapy was conducted on the Ethos system. The first five fractions were delivered in conventional image-guided radiotherapy (IGRT) mode, whereas the remaining 23 fractions were delivered in the Ethos online ART mode.

Imaging data acquisition and processing

For each online ART treatment session, the patient took two iCBCT scans. The first scan (iCBCT_1_) was performed after positioning and was used for online recontouring and replanning. After the beam delivery, the second scan (iCBCT_2_) was performed to verify the patient's intrafraction motion during the online ART process. For retrospective analysis, the online session iCBCT images were imported into Eclipse (Varian Medical Systems, Palo Alto, CA). The regular CTVu were contoured separately on both iCBCT_1_ and iCBCT_2_ by a certificated gynecological oncologist with 10 years of experience. The regular CTV contouring was guided by the Radiation Therapy Oncology Group guideline [21], including lymph nodes, uterus, parametria, cervix, and vaginal tissues. Before processing, the iCBCT images and associated contours were rigidly registered and resampled to 1 x 1 x 1 mm3 to avoid possible dimension mismatching or voxel size differences.

Personalized margin search algorithm

**Figure 1:**
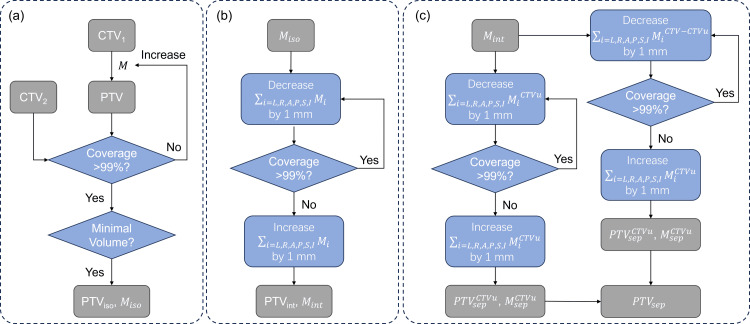
The workflow for the personalized margin search algorithm. (a) The initial search for an isotropic margin as the starting point. (b) The search for an anisotropic margin of the integral CTV1. (c) The search for anisotropic margins of CTVu and CTV-CTVu, respectively CTV: clinical target volume; CTV_1_: clinical target volume in the first iterative cone-beam computed tomography scan; CTV_2_: clinical target volume in the second iterative cone-beam computed tomography scan; CTVu: uterus part of CTV; PTV: planning target volume; L: left; R: right; A: anterior; P: posterior; S: superior; I: inferior; M: margin; iso: isotropic; int: integral; sep: separate

The registered contours were converted to 3D binary masks via MATLAB (The MathWorks, Inc., Natick, MA) for processing. The contour margin expansion in different directions was achieved by volumetric convolution between the original contour and an anisotropic kernel. The personalized margin search algorithm is demonstrated in Figure 1. First, the CTV in iCBCT_1_ (CTV_1_) was expanded isotropically to find all possible margin values that could cover 99% of the CTV (CTV_2_) in iCBCT_2_. After the initial search, the margin values that gave the smallest volume were recorded as $M_{iso}$, which could be mathematically expressed as

$M_{iso} = [L_{iso}, R_{iso}, A_{iso}, P_{iso}, S_{iso}, I_{iso}]$ (1)

where $L_{iso}$, $R_{iso}$, $A_{iso}$, $P_{iso}$, $S_{iso}$, and $I_{iso}$ represent the margin values in different directions and they were all equal.

The isotropic $M_{iso}$ was a starting point for further anisotropic margin search. To find a minimal margin, the margin value in all six directions, i.e., $L_{iso}$, $R_{iso}$, $A_{iso}$, $P_{iso}$, $S_{iso}$, and $I_{iso}$, was reduced iteratively until the resulting PTV_iso_ covered less than 99% of CTV_2_ or the margin value on a certain direction reached 1 mm. The final margin value for the integral CTV_1_ was recorded as $M_{int}$, and

$M_{int} = [L_{int}, R_{int}, A_{int}, P_{int}, S_{int}, I_{int}]$. (2)

In addition to the integral margin, the separate margins for the uterus (CTVu) and the remaining CTV (CTV-CTVu) were also searched using a similar strategy. The margin for CTVu was reduced from $M_{int}$ iteratively until the CTV_2_ coverage was smaller than 99% or the margin value in a certain direction reached 1 mm, and the value was recorded as $M_{sep}^{CTVu}$. The margin for CTV-CTVu was searched in the same way and recorded as $M_{sep}^{CTV-CTVu}$. Consequently,

$M_{sep}^{CTVu} = [L_{sep}^{CTVu}, R_{sep}^{CTVu}, A_{sep}^{CTVu}, P_{sep}^{CTVu}, S_{sep}^{CTVu}, I_{sep}^{CTVu}]$ (3)

$M_{sep}^{CTV-CTVu} = [L_{sep}^{CTV-CTVu}, R_{sep}^{CTV-CTVu}, A_{sep}^{CTV-CTVu}, P_{sep}^{CTV-CTVu}, S_{sep}^{CTV-CTVu}, I_{sep}^{CTV-CTVu}]$. (4)

Implementation and evaluation metrics

The first several online ART sessions were monitored to find an optimal margin value for CTV1 that could cover more than 99% of CTV2 with the smallest possible volume. $M_{int}$, $M_{sep}^{CTVu}$, and $M_{sep}^{CTV-CTVu}$​​​​ were found for the sessions and the maximal values were applied for the following sessions for evaluation. Consequently, two PTVs were generated and compared:

$PTV_{int} = M_{int} \cdot CTV_1$ (5)

$PTV_{sep}^{CTVu} = M_{sep}^{CTVu} \cdot CTVu$ (6)

$PTV_{sep}^{CTV-CTVu} = M_{sep}^{CTV-CTVu} \cdot (CTV-CTVu)$ (7)

$PTV_{sep} = PTV_{sep}^{CTV} + PTV_{sep}^{CTV-CTVu}$. (8)

The evaluation metrics for $PTV_{int}$ and $PTV_{sep}$ include CTV_2_ coverage and absolute volume.

Results

The patient completed 23 online ART sessions, and the average time between two iCBCT scans was 26.1 ± 6.1 minutes. The patient did not have sufficient rectum preparation in the first session, and a large deformation existed between iCBCT_1_ and iCBCT_2_. The oncologist decided not to treat that session with the adapted plan but with the scheduled plan. Therefore, the first session was excluded from data analysis.

**Figure 2:**
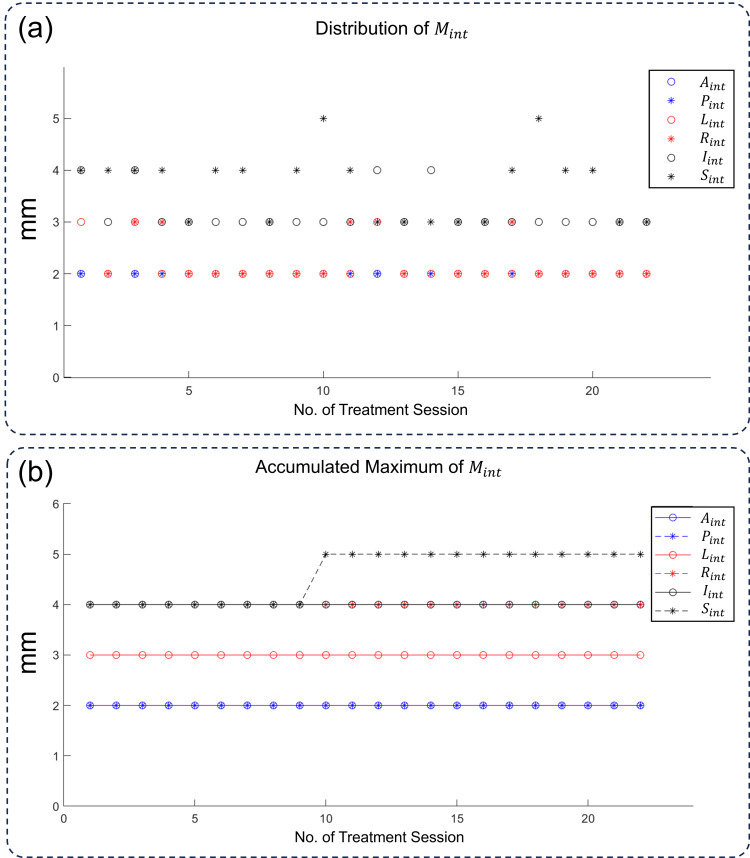
The changes in the integral margin for CTV in all sessions. (a) The distribution of Mint in all 22 ART sessions. (b) The accumulated maximum of Mint, which is the maximum value of Mint from the first to the nth fraction L: left; R: right; A: anterior; P: posterior; S: superior; I: inferior; M: margin; int: integral; CTV: clinical target volume; ART: adaptive radiation therapy

The distribution of $M_{int}$ was plotted in Figure 2a. As shown, the distribution of margins in all directions fell into small individual ranges, indicating that the patient's situation was steady throughout the treatment course. The mean ± SD values for $L_{int}$, $R_{int}$, $A_{int}$, $P_{int}$, $S_{int}$, and $I_{int}$ are 2.00 ± 0.00, 2.00 ± 0.00, 2.14 ± 0.36, 2.38 ± 0.59, 3.19 ± 0.40, and 3.71 ± 0.64 mm, respectively. Figure 2b shows the accumulated maximum of $M_{int}$, i.e., the maximal margin values in the first n sessions. Since $M_{int}$ varied only in small ranges, the accumulated maximum of $M_{int}$ saturated quickly and almost kept constant.

**Figure 3:**
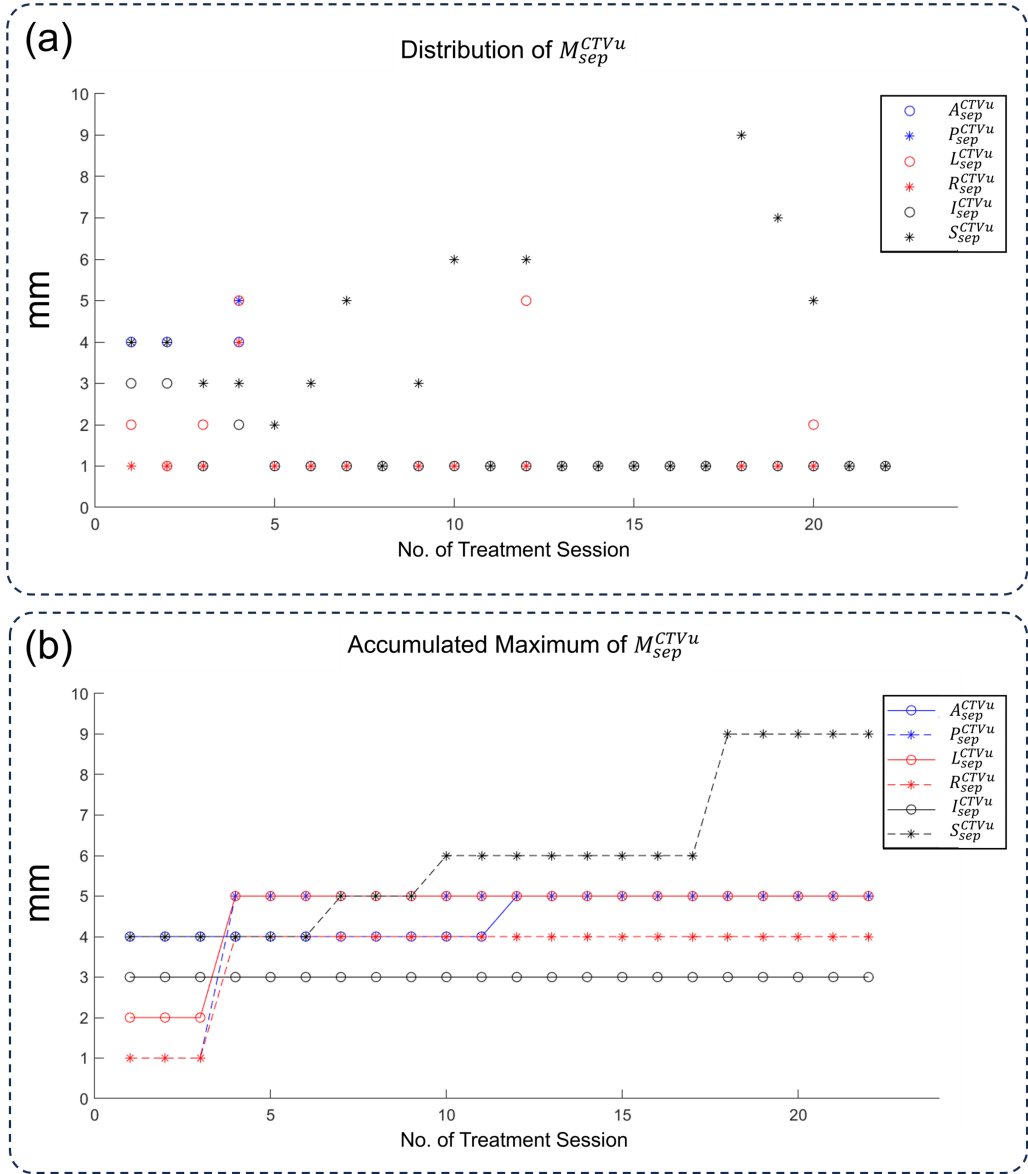
The changes in margin for CTVu in all sessions. (a) The distribution of $M_{sep}^{CTVu}$ in all 22 ART sessions. (b) The accumulated maximum of $M_{sep}^{CTVu}$ , which is the maximum value of $M_{sep}^{CTVu}$ from the first to the nth fraction CTV: clinical target volume; CTVu: uterus part of CTV; L: left; R: right; A: anterior; P: posterior; S: superior; I: inferior; M: margin; sep: separate

The distribution of $M_{sep}^{CTVu}$ was plotted in Figure 3a, which covered a wider range of magnitudes than those in Figure 2. The mean ± SD values for $L_{sep}^{CTVu}$, $R_{sep}^{CTVu}$, $A_{sep}^{CTVu}$, $P_{sep}^{CTVu}$, $S_{sep}^{CTVu}$, and $I_{sep}^{CTVu}$ are 1.62 ± 1.32, 1.19 ± 0.87, 1.52 ± 1.21, 1.14 ±, 0.65, 1.24 ± 0.62, and 3.24 ± 2.36 mm, respectively. Figure 3b displays the accumulated maximum of $M_{sep}^{CTVu}$. The curve increased as more sessions were included, and the saturation was also slower as some outliers existed in the latter sessions.

**Figure 4:**
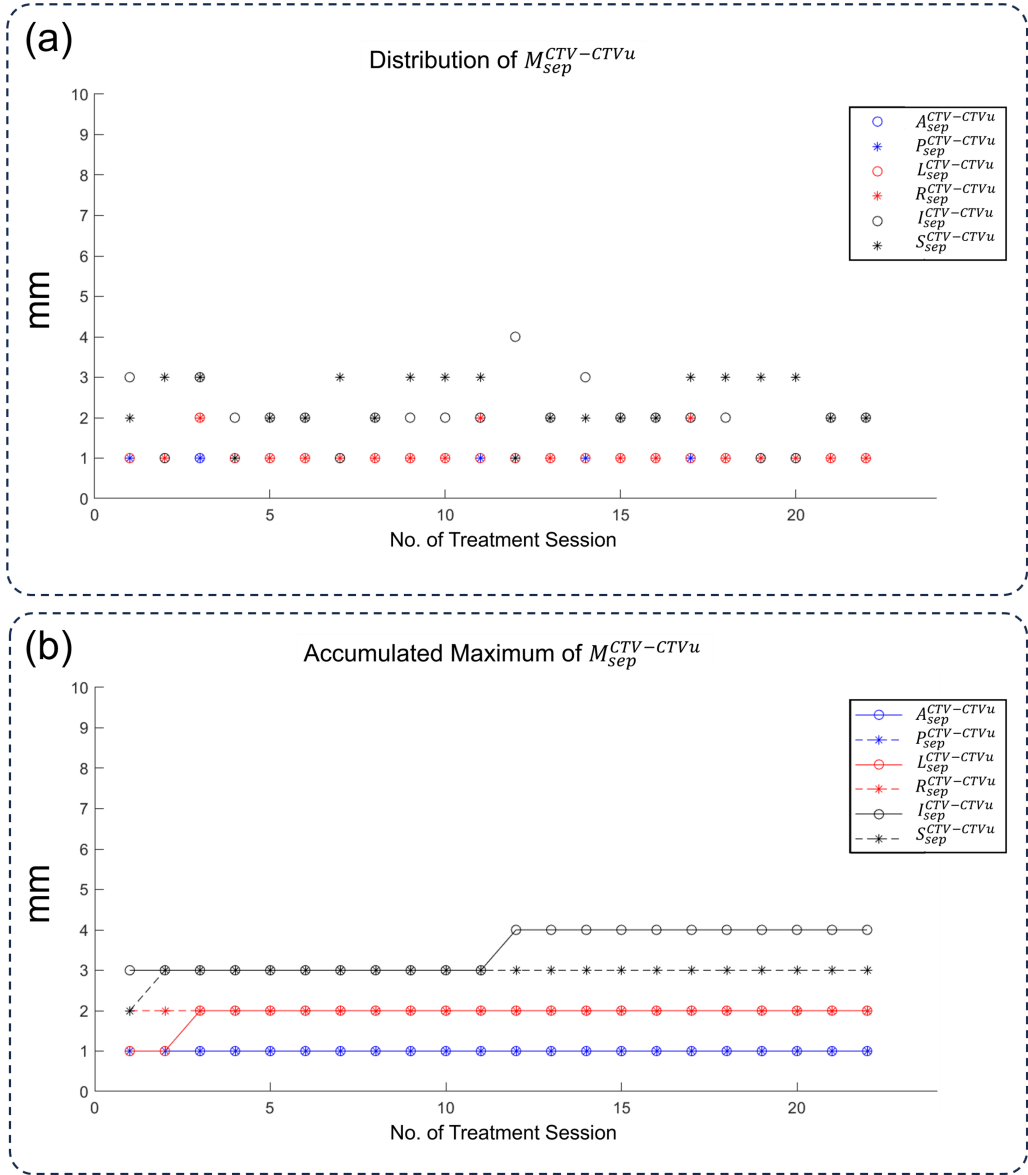
The changes in the margin for the remaining CTV. (a) The distribution of $M_{sep}^{CTV-CTVu}$ in all 22 ART sessions. (b) The accumulated maximum of $M_{sep}^{CTV-CTVu}$ , which is the maximum $M_{sep}^{CTV-CTVu}$ value from the first to the nth fraction CTV: clinical target volume; CTVu: uterus part of CTV; L: left; R: right; A: anterior; P: posterior; S: superior; I: inferior; M: margin; sep: separate

The distribution of $M_{sep}^{CTV-CTVu}$ was plotted in Figure 4a, and the margin values in all directions fell into small individual margins, similar to $M_{int}$. The mean ± SD values for $L_{sep}^{CTV-CTVu}$, $R_{sep}^{CTV-CTVu}$, $A_{sep}^{CTV-CTVu}$, $P_{sep}^{CTV-CTVu}$​​​​​​​, $S_{sep}^{CTV-CTVu}$​​​​​​​, and $I_{sep}^{CTV-CTVu}$​​​​​​​ are 1.00 ± 0.00, 1.00 ± 0.00, 1.05 ± 0.22, 1.24 ± 0.44, 2.05 ± 0.74, and 2.38 ± 0.67 mm, respectively. Figure 4b shows the accumulated maximum of $M_{sep}^{CTV-CTVu}$, which saturated quickly since the variation of it was small. As shown, the margin values for $M_{sep}^{CTV-CTVu}$​​​​​​​​​​​​​​ were significantly smaller than those of $M_{sep}^{CTVu}$​​​​​​​​​​​​​​.

**Figure 5:**
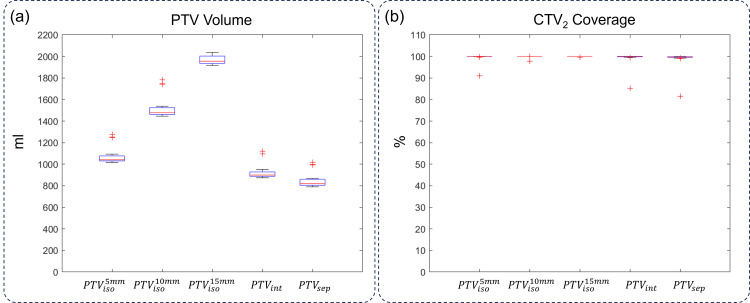
Evaluation results of different margins. (a) PTV volume comparison of different margin strategies. (b) CTV2 coverage of different margin strategies CTV_2_: clinical target volume in the second iCBCT scan; PTV: planning target volume; iso: isotropic; int: integral; sep: separate

Based on the margin value distribution and saturation speed, n = 5 was determined to be the number of monitored sessions, and the accumulated maximum of $M_{int}$, $M_{sep}^{CTVu}$, and $M_{sep}^{CTV-CTVu}$​​​​​​​ was applied for all sessions for PTV volume and CTV_2_ coverage comparison, and the results were shown in Figure 5. Also, isotropic margins of 5, 10, and 15 mm were included as comparison baselines. As shown in Figure 5a, the PTV volume increased rapidly with larger isotropic margins, as expected. By contrast, $PTV_{int}$​​​​​​​​​ ​​​​​and $PTV_{sep}$​​​​​​​​​​​​​​​​​​​​​ gave smaller volumes as $M_{int}$​​​​​​​ and $M_{sep}$​​​​​​​ were both smaller than 5 mm in all directions. The mean ± SD values for $PTV_{iso}^{5mm}$​​​​​​​​​​​​​​, $PTV_{iso}^{10mm}$​​​​​​​, $PTV_{iso}^{15mm}$​​​​​​​, $PTV_{int}$​​​​​​​, and $PTV_{sep}$​​​​​​​ were 1,074.0 ± 78.1, 1,519.5 ± 100.4, 2,006.4 ± 122.5, 929.3 ± 73.4, and 845.1 ± 72.5 mL, respectively. The volume difference between $PTV_{int}$ and $PTV_{sep}$​​​​​​​ was significant (p < 0.001). As for CTV_2_ coverage, all the strategies ensured an average coverage larger than 99%, and the differences between any two strategies were insignificant.

Table 1 lists the dosimetric comparison of major OARs in plans with different margins, including $M_{iso}^{5mm}$, $M_{int}$, and $M_{sep}$. The results showed that compared to the isotropic 5 mm margin, the proposed personalized anisotropic margin significantly reduced OAR doses. For example, the V40 of the rectum decreased by 9%, the V30 of the small intestine decreased by 13.8%, and the volume of the body receiving the prescription dose (V50.4) decreased by 14.7%.

**Table 1:** OAR dose comparison between different margins OAR: organs at risk; M: margin; iso: isotropic; int: integral; sep: separate; L: left; R: right

OAR	Metric	M_iso_^5mm^	M_int_	M_sep_
Rectum	V40 (%)	43.3	44.3	39.4
	D2cc (cGy)	5,271.2	5,186	5,169
	Mean (cGy)	3,609.0	3,518.7	3,375.2
Bladder	V40 (%)	34.8	34.5	32.9
	D2cc (cGy)	5,310.3	5,346.3	5,293.8
	Mean (cGy)	2,797.8	2,823.1	2,712.9
Femur head (L)	V40 (%)	0.3	1.9	0.1
	Mean (cGy)	1,629.0	1,753.3	1,643.8
Femur head (R)	V40 (%)	2.5	3.7	1.1
	Mean (cGy)	1720.6	1772.6	1697.8
Small intestine	V30 (%)	67.9	61.7	58.5
	V50 (%)	7.2	6.9	5.8
	Mean	1,862.2	1,909.6	1,808.9
Marrow	V30 (%)	99.4	112.4	101.1
	D90 (cGy)	1,257.9	1,276.5	1,233.0
	Mean	2,832.4	2,943.8	2,726.6
Body	V50.4 (mL)	1,201.2	1,194.2	1,024.1
	V30 (mL)	2,788.4	2,859.9	2,588.3

## Discussion

In this study, we developed a novel personalized anisotropic margin search algorithm for cervical cancer RT. The online ART technique enabled by the Ethos system eliminates interfraction uncertainties as treatments are adapted to the patient's daily anatomy and positioning. However, intrafraction motions still exist and could become even more severe than conventional IGRT as patients spend more time in each treatment session. To our knowledge, it is the first study developing methods to find personalized anisotropic margins to address intrafraction motions in iCBCT-based online cervical cancer treatment. This method holds great potential for reducing PTV volume and lessening normal tissue complications while maintaining CTV coverage.

In conventional IGRT, the CTV-to-PTV margin is at least 5 mm and sometimes 10 mm in conservative scenarios. Although these margins ensure CTV coverage, many surrounding normal tissues are also included in PTV. With personalized anisotropic margin, however, PTV volume decreases from 1,074.0 ± 78.1 and 1,519.5 ± 100.4 mL to 929.3 ± 73.4 mL in $PTV_{int}$, or by 13.5% and 38.8%, respectively. If CTVu was considered separately, PTV volume can further decrease to 845.1 ± 72.5 mL or by 21.3% and 44.3%, respectively. The volume reduction can spare normal tissue better and potentially reduce the OAR doses and complications.

As shown in Figures 3, 4, the margin values for $M_{sep}^{CTV-CTVu}$ were significantly smaller than those of $M_{sep}^{CTVu}$, which was also reflected in the smaller volume of $PTV_{sep}$ compared to $PTV_{int}$. This indicated that the uterus contributed primarily to intrafraction motions and deserved to be considered separately. Although many studies have shown that CTV-to-PTV margins could be smaller in online ART systems [10,12], the long treatment time may cause more bladder filling and intestinal motility, increasing intrafraction in certain directions [22].

There are also limitations in this study. First, this study enrolled only one case as an initial experiment, and more subjects will be included in future studies to verify the personalized anisotropic margin search algorithm further. Second, this study only searched physical margins and did not do dosimetric evaluations. However, PTV volume and CTV coverage results revealed that target coverage could be maintained. The target volume was significantly reduced with the personalized anisotropic margin, and less dose to OARs and full prescription dose to targets are expected. We will conduct dosimetric experiments and report the results in future studies.

## Conclusions

In this study, we proposed a novel personalized anisotropic margin search algorithm for cervical cancer RT. Compared to isotropic 5 or 10 mm margin, the personalized anisotropic margin reduced PTV volume from 1,074.0 ± 78.1 and 1,519.5 ± 100.4 mL to 929.3 ± 73.4 mL in $PTV_{int}$, or by 13.5% and 38.8%, respectively; if the uterus was considered separately, the volume can be further reduced to 845.1 ± 72.5 mL in $PTV_{sep}$, or by 21.3% and 44.3%, respectively, while CTV coverage was still maintained. This algorithm could reduce target volume and potentially spare normal tissue better than isotropic margin expansion.
